# Supplementary material for: Storage and disposal practices of unused and expired medications among adult women attending Banadir Hospital, Mogadishu, Somalia: A cross-sectional study
Source: PLoS One. 2026 May 4;21(5):e0348446. doi: 10.1371/journal.pone.0348446 (PMC13138632; doi:10.1371/journal.pone.0348446)
Supplement: S1_File — This file contains all the questions used for this study. (PDF) [file pone.0348446.s001.pdf]

S1 Appendix. Questionnaire used for data collection

Storage and disposal practices of unused and expired medications among adult women attending Banadir Hospital, Mogadishu, Somalia: A cross-sectional study

Introduction

This questionnaire is part of a research study titled "Storage and disposal practices of unused and expired medications among adult women attending Banadir Hospital, Mogadishu, Somalia: A cross-sectional study." The aim of this survey is to assess household practices related to the storage and disposal of unused and expired medications. The information obtained will help identify current practices and inform strategies to improve safe medication management and public health interventions. Participation is voluntary, and all responses will remain confidential and used only for research purposes.

Please answer the questions honestly by ticking (✓) the most appropriate response.

Section A. Socio-demographic characteristics

1. Age (years) ___________

2. Marital status

☐ Married
☐ Unmarried

3. Education level

☐ No formal education
☐ Primary
☐ Intermediate
☐ Secondary
☐ University

4. Occupation

☐ Employed
☐ Unemployed

5. Monthly income (USD)

☐ ≤100
☐ 101–200
☐ 201–300

☐ 301–400
☐ >400

Section B. Storage and disposal practices of unused medications

6. **Do you currently have unused medication at home?**

☐ Yes
☐ No

7. If yes, why do you keep unused medication? (If you answered "Yes" to Question 6)

☐ For future use
☐ For emergency use
☐ Share with friends or family members
☐ Other (please specify) __________

8. Are you familiar with the proper methods for storing medications at home?

☐ Yes
☐ No

9. Do you read the storage directions provided on the labels or leaflets?

☐ Yes
☐ No

10. Where do you keep the unused medication?

☐ Kitchen cabinet
☐ Bathroom cabinet
☐ Bedroom cabinet
☐ Medicine box
☐ Refrigerator
☐ Other (please specify) __________

11. Do you dispose of unused medications?

☐ Yes
☐ No

12. How do you dispose of unused medications?

☐ Discard in household trash
☐ Share with friends or family members
☐ Return to a pharmacy
☐ Return to a hospital or clinic
☐ Other (please specify) __________

Section C. Storage and disposal practices of expired medications

13. Do you currently have expired medication at home?

☐ Yes
☐ No
☐ I don't know

14. If yes, why do you keep expired medication? (If you answered "Yes" to Question 13)

☐ I do not have time to sort through them
☐ I forgot they were there
☐ I do not consider them dangerous
☐ I was planning to dispose of them anyway
☐ I don't know

15. Do you check the expiration date before purchasing a medication?

☐ Yes
☐ No

16. Do you check the expiration date before using a medication?

☐ Yes
☐ No

17. Are you familiar with the proper method for disposing of a nearly expired medication?

☐ Yes
☐ No

18. What method do you consider appropriate for disposing of a nearly expired medication?

☐ Discard in household trash
☐ Return to a hospital or clinic
☐ Keep at home until expired

☐ Return to a pharmacy
☐ Flush in toilet or sink
☐ Share with friends or family members
☐ Other (please specify) __________

19. Are you familiar with the proper method for disposing of expired medications?

☐ Yes
☐ No

20. What is the proper method for disposing of expired medications?

☐ Discard in household trash
☐ No action (keep at home)
☐ Return to a pharmacy or hospital
☐ Flush in toilet or sink
☐ Other (please specify) __________
