# Supplementary material for: Storage and disposal practices of unused and expired medications among adult women attending Banadir Hospital, Mogadishu, Somalia: A cross-sectional study
Source: PLoS One. 2026 May 4;21(5):e0348446. doi: 10.1371/journal.pone.0348446 (PMC13138632; doi:10.1371/journal.pone.0348446)
Supplement: Attachment — Submitted filename: Response to Reviewers.pdf [file pone.0348446.s004.pdf]

Author Responses

Submitting Author: Abdiweli Mohamed Abdi

Date: 18 March 2026

Subject: Submission of the corrected manuscript

Dear Editor,

We would like to extend our sincere gratitude to you and the reviewers for your valuable and constructive comments, suggestions, and questions. Please find attached our point-by-point response letter addressing the comments and suggestions on the manuscript entitled **"Storage and disposal practices of unused and expired medications among adult women attending Banadir Hospital, Mogadishu, Somalia: A cross-sectional study."**

We have carefully revised the manuscript in response to the editor's and reviewers' comments and suggestions. We also confirm that the correct Funding Statement is: **"The authors received no specific funding for this work."** In addition, we have uploaded the required files separately as **Response to Reviewers**, **Revised Manuscript with Track Changes**, and **Manuscript**.

We hope that the revised version of our manuscript is now suitable for publication in your esteemed journal, and we look forward to your favorable consideration.

**Academic Editor**		
**Comment/Suggestions**	**Author's Response**	**Position**
**1. Comment:**<br><br>Please ensure that your manuscript meets PLOS ONE's style requirements, including those for file naming.	***Response:***<br>*Thank you for this important comment. We carefully revised the manuscript to align with PLOS ONE formatting requirements using the journal's main body and title/authors/affiliations templates. We also standardized the manuscript structure, title page, author affiliations, headings, abstract, keywords, and end sections to match PLOS ONE style. In addition, the*	Not applicable

	submission files were renamed according to the journal's instructions and uploaded separately as: Response to Reviewers, Revised Manuscript with Track Changes, and Manuscript.	
**2. Comment:**<br><br>In the ethics statement in the Methods, you have specified that verbal consent was obtained. Please provide additional details regarding how this consent was documented and witnessed, and state whether this was approved by the IRB.	***Response:***<br>*Thank you for this important comment. We have revised the ethics statement in the Methods section to clarify the verbal consent process. Specifically, we added that the study objectives and procedures were explained to each participant in the Somali language before participation, and that after participants verbally agreed to participate, the data collectors documented consent by marking a designated consent checkbox on the questionnaire form. We also clarified that the consent process was conducted in the presence of the trained interviewer, who served as a witness. In addition, we explicitly stated that the Research Ethics Committee (REC) of Al Hayat Medical University approved the use of verbal informed consent for this study.*	Page (s): 7-8<br>Line (s): 151-159
**3. Comment:**<br>Thank you for stating the following in the Acknowledgments Section of your manuscript: "None" We note that you have provided funding information that is not currently declared in your Funding Statement. However, funding information should not appear in the Acknowledgments section or	***Response:***<br>*Thank you for this clarification. We would like to respectfully note that we did not report receipt of any funding in the Acknowledgments section or elsewhere in the manuscript. There may have been a misunderstanding regarding this point. Our intended and correct Funding Statement is: "The authors received no specific funding for this work." We confirm that no funding-related text has been included in the Acknowledgments section, and we request that*	Page (s): 24<br>Line (s): 362

other areas of your manuscript. We will only publish funding information present in the Funding Statement section of the online submission form. Please remove any funding-related text from the manuscript and let us know how you would like to update your Funding Statement. Currently, your Funding Statement reads as follows: "The author(s) received no specific funding for this work."	*the online submission form retain or be updated to reflect the following Funding Statement exactly: "The authors received no specific funding for this work."*	
**4. Comment:**<br>We note that your Data Availability Statement is currently as follows: "All relevant data are within the manuscript and its Supporting Information files." Please confirm at this time whether or not your submission contains all raw data required to replicate the results of your study. Authors must share the minimal data set for their submission.	***Response:***<br>*Thank you for this important comment. We confirm that the submission contains the complete dataset required to replicate the findings of this study. Specifically, all relevant data necessary to reproduce the statistical analyses and reported results have been provided as Supporting Information in S2 File (xlsx).*<br><br>*We have revised the **Data Availability Statement** to read as follows: "All relevant data required to replicate the findings of this study are provided in Supporting Information as S2 File (xlsx)."*<br><br>*We have also clarified the **Supporting Information section** as follows: "S1 File. Questionnaire. This file contains all the questions used for this study. (pdf)" and "S2 File. Dataset. This file contains the complete*	Page (s): 24<br>Line (s): 366-367<br><br>Page (s): 30<br>Line (s): 508-511

	dataset necessary to replicate the statistical analyses and findings reported in this study. (xlsx).".	
**5. Comment:**<br><br>Your ethics statement should only appear in the Methods section of your manuscript. If your ethics statement is written in any section besides the Methods, please delete it from any other section.	***Response:***<br>*Thank you for this comment. We have revised the manuscript accordingly and confirmed that the ethics statement now appears only in the Methods section.*	Page (s): 7-8<br>Line (s): 147-159
**6. Comment:**<br>If the reviewer comments include a recommendation to cite specific previously published works, please review and evaluate these publications to determine whether they are relevant and should be cited. There is no requirement to cite these works unless the editor has indicated otherwise.	***Response:***<br>*Thank you for this clarification. We carefully reviewed the comments from both reviewers and confirm that we did not receive any recommendations to cite specific previously published works.*	Not applicable.
**7. Comment:**<br>The manuscript should better justify why this sampling method	***Response:***<br>*Thank you for this important comment. We have revised the Methods section to provide a clearer*	Page (s): 5<br>Line (s): 88-96

was selected, discuss potential selection bias, and explain why it only included individuals visiting the hospital pharmacy.	*justification for the use of consecutive sampling and the selection of the hospital outpatient pharmacy as the recruitment site. Specifically, we clarified that consecutive sampling was chosen because it allows inclusion of all accessible eligible participants in a clinical setting and is commonly used in hospital-based cross-sectional studies where a complete sampling frame is not available. We also explained that the outpatient pharmacy was selected because it is the point at which patients obtain prescribed medications, making it an appropriate setting for assessing medication storage and disposal practices. In addition, we acknowledged the potential for selection bias by stating that this approach includes only individuals visiting the outpatient pharmacy and may therefore limit representativeness of all patients visiting other departments of the hospital.*	
**8. Comment:**<br>Revise the discussion part; it would benefit from a deeper exploration of contextual factors specific to Somalia.	***Response:***<br>*Thank you for this valuable comment. We have revised the Discussion to include a broader interpretation of the findings within the specific context of Somalia's health system and pharmaceutical governance environment. In particular, we added discussion on the structural challenges affecting the Somali health sector, including weak regulatory oversight, fragmented service delivery, and the heavy reliance on private health providers and pharmacies for access to medicines. We also incorporated recent national and international policy developments, including the need to strengthen pharmaceutical governance and the establishment of the Interim National Medicines*	Page (s): 22<br>Line (s): 310-323

	Regulatory Authority. These additions were intended to better situate our findings within the Somali context and to explain how health system factors may influence household medication storage and disposal practices.	
**9. Comment:**<br>Provide additional information on questionnaire validation; the complete questionnaire should be included in an appendix to improve clarity and transparency.	***Response:***<br>*Thank you for this valuable comment. We have revised the Methods section to provide additional information on questionnaire development and validation. Specifically, we clarified that the questionnaire was developed based on previously published studies, reviewed by two public health experts with experience in pharmaceutical practice to support content validity, and pretested among 43 respondents who were not included in the main study to assess clarity, relevance, and comprehensibility. We also added the internal consistency result of the instrument, with a Cronbach's alpha of 0.790, indicating acceptable reliability. In addition, to improve clarity and transparency, we have included the complete questionnaire as S1 File in the Supporting Information section.*	Page (s): 6<br>Line (s): 109-129
Reviewer #1		
**Reviewer's Comment**	**Author's Response**	**Position**
**1. Comment:**<br>The abstract contains a large amount of numerical detail, which makes it dense. Streamlining the abstract to highlight the most important findings and implications would improve clarity.	***Response:***<br>*Thank you for this helpful comment. We revised the abstract to improve clarity and reduce excessive numerical detail while retaining the most important findings. Specifically, we streamlined the presentation of the results by focusing on the key patterns observed in medication storage, label reading, expiration-date*	Page (s): 2<br>Line (s): 31-39

	checking, and disposal practices, and we retained only the most relevant numerical information needed to summarize the main findings. We also emphasized the overall implication that improper medication storage and disposal practices were highly prevalent in this setting and highlighted the need for public awareness programs and the establishment of a national drug take-back system.	
**2. Comment:**<br>The study uses consecutive sampling at a single hospital outpatient pharmacy. While this is reasonable in the given context, it limits the generalizability of the findings. This should be more clearly acknowledged in the Discussion and Limitations sections, and conclusions should be phrased cautiously.	***Response:***<br>*Thank you for this important comment. We have revised the manuscript to acknowledge this limitation more explicitly in the Discussion, Limitations and strengths, and Conclusion sections.*<br><br>***In the Discussion:***<br>*We now state that the findings should be interpreted with caution because the study used consecutive sampling and was conducted among women attending a single hospital outpatient pharmacy in Mogadishu, which may limit applicability to other populations and settings in Somalia.*<br><br>***In the Limitations and strengths section:***<br>*We further clarify that the use of consecutive sampling at a single outpatient pharmacy may introduce selection bias and limit the generalizability of the findings to other populations, healthcare settings, and regions of Somalia, and that including only adult women attending this facility may not fully represent medication storage and disposal practices in the wider community.*	 <br><br><br><br><br>Page (s): 21-22<br>Line (s): 306-309<br><br><br><br><br><br><br>Page (s): 22-23<br>Line (s): 330-334

	In the Conclusion section:<br>*We also revised the Conclusion section to use more cautious language.*	Page (s): 23<br>Line (s): 341-345
**3. Comment:**<br>The manuscript frequently refers to "proper" storage and disposal practices; however, Somalia currently lacks national guidelines for medication disposal. The authors should clearly define what they consider "proper" practices and indicate whether these are based on WHO or other international recommendations.	***Response:*** *Thank you for this important comment. We have revised the Introduction to clearly define what is meant by "proper" medication storage and disposal practices in the context of this study.*<br>*These definitions are based on international guidance and recommendations, given the current absence of national guidelines for medication storage and disposal in Somalia.*	Page (s): 3<br>Line (s): 45-48
**4. Comment:**<br>A large proportion of participants reported being familiar with proper disposal methods, yet most identified household trash as the correct method. This apparent inconsistency suggests misconceptions rather than true knowledge and should be explicitly discussed.	***Response:***<br>*Thank you for this valuable comment. We have revised the Discussion to explicitly address the inconsistency between participants' self-reported familiarity with proper disposal methods and their identification of household trash as the main disposal method for unused, nearly expired, and expired medications. We now clarify that this pattern likely reflects misconceptions rather than a correct understanding of recommended disposal practices and highlights a clear gap between perceived awareness and actual knowledge of proper medication disposal.*	Page (s): 20<br>Line (s): 279-284
**5. Comment:**<br>More information is needed on how key terms such as "unused	***Response:***<br>*Thank you for this important comment. We have revised the Data collection instrument and procedure*	Page (s): 6<br>Line (s): 123-127

medication" and "expired medication" were explained to participants during the interviews, to ensure consistent understanding.	*section to clarify how these key terms were explained during the interviews. Specifically, we added that data collectors explained that "unused medication" referred to any medication that had been obtained but was not fully consumed or was no longer intended for use, while "expired medication" referred to any medication that had passed the expiry date indicated on its packaging or label.*	
**6. Comment:**<br>The use of chi-square tests and Cramer's V is appropriate. However, the manuscript describes some associations as "strong" or "moderate" without specifying the thresholds used. The authors should define these categories or use more neutral wording.	***Response:***<br>*We have revised the Data analysis section to define the thresholds used for interpreting the magnitude of associations based on Cramér's V. Specifically, we added that, for statistically significant chi-square tests ($p < 0.05$), Cramér's V was interpreted as weak (≤0.20), moderate (>0.20 to ≤0.60), or strong (>0.60), based on the IBM Cognos Analytics documentation on Cramér's V in 2025.*	Page (s): 7<br>Line (s): 144-146
**7. Comment:**<br>The Discussion section is well referenced but occasionally repeats results already presented earlier. Reducing repetition and focusing more on interpretation and implications would strengthen this section.	***Response:***<br>*Thank you for this helpful comment. We revised the Discussion section to reduce repetition of results and place greater emphasis on interpretation and implications. In the revised version, numerical details were removed where they only repeated findings already presented in the Results section and were retained only where needed for comparison with previous studies. We also revised the wording throughout the Discussion to make it more interpretive and to better highlight the public health and contextual implications of the findings.*	Page (s): 18-22<br>Line (s): 232-323

**8. Comment:**<br>The manuscript would benefit from careful language and formatting revision to address minor grammatical errors, spacing issues, and inconsistencies throughout the text.	***Response:***<br>*Thank you for this helpful comment. We carefully revised the manuscript to improve language clarity and consistency throughout the text. Specifically, we corrected minor grammatical errors, spacing issues, and formatting inconsistencies to improve readability and overall presentation.*	Throughout the manuscript.

Reviewer #2		
**Reviewer's Comment**	**Author's Response**	**Position**
**Comment:**<br>Overall, the manuscript is well written and clearly structured. The study appears to be methodologically sound, and the authors demonstrate careful attention to detail, particularly in the data collection process, which supports the reliability of the findings.	**Response:**<br>We sincerely thank the reviewer for the positive and encouraging assessment of our manuscript. We appreciate the recognition of the study's methodological approach, the care taken in the data collection process, and the relevance of the topic within the local context. We are also grateful for the reviewer's acknowledgment that the findings may help inform targeted programs and awareness initiatives for the community.	Not applicable; no revision requested.
